# Modelling of human cooling in cold water: effect of immersion level

**DOI:** 10.1186/2046-7648-4-S1-A132

**Published:** 2015-09-14

**Authors:** Irena Yermakova, Anastasiia Nikolaienko, Yuliia Solopchuk, Michael Regan

**Affiliations:** 1 International Scientific-Training Centre for Information Technologies and Systems, National Academy of Sciences, Kiev, Ukraine; 2 National University for Physical Activity and Sport, Kiev, Ukraine

## Introduction

Cold water immersion is a severe challenge for humans. Mathematical modelling of human thermoregulatory responses is an alternative approach to study cooling in water. Preliminary modelling prediction can be a useful tool for preventive steps that will help to decrease or even to avoid health hazard [1]. The purpose of this study was to model core cooling rates in cold water for some immersion levels in humans.

## Methods

The complexity of multi-compartmental models for human thermoregulation is realised as Information Technology (IT) in Borland C++ Builder 2010 [2]. IT is a suitable tool that allows user to input individual data, water and air parameters, immersion level and physical exercise. Output data represent the dynamics of all local temperatures, muscles and skin blood flows, shivering, water convection, heat internal flows, heat losses, etc. Three levels of the human immersion in cold water were simulated: #1: whole body immersion; #2: head out of water; #3: head, arms and hands out of water. Water and air temperatures were 10 °C.

## Results

**Figure 1:**
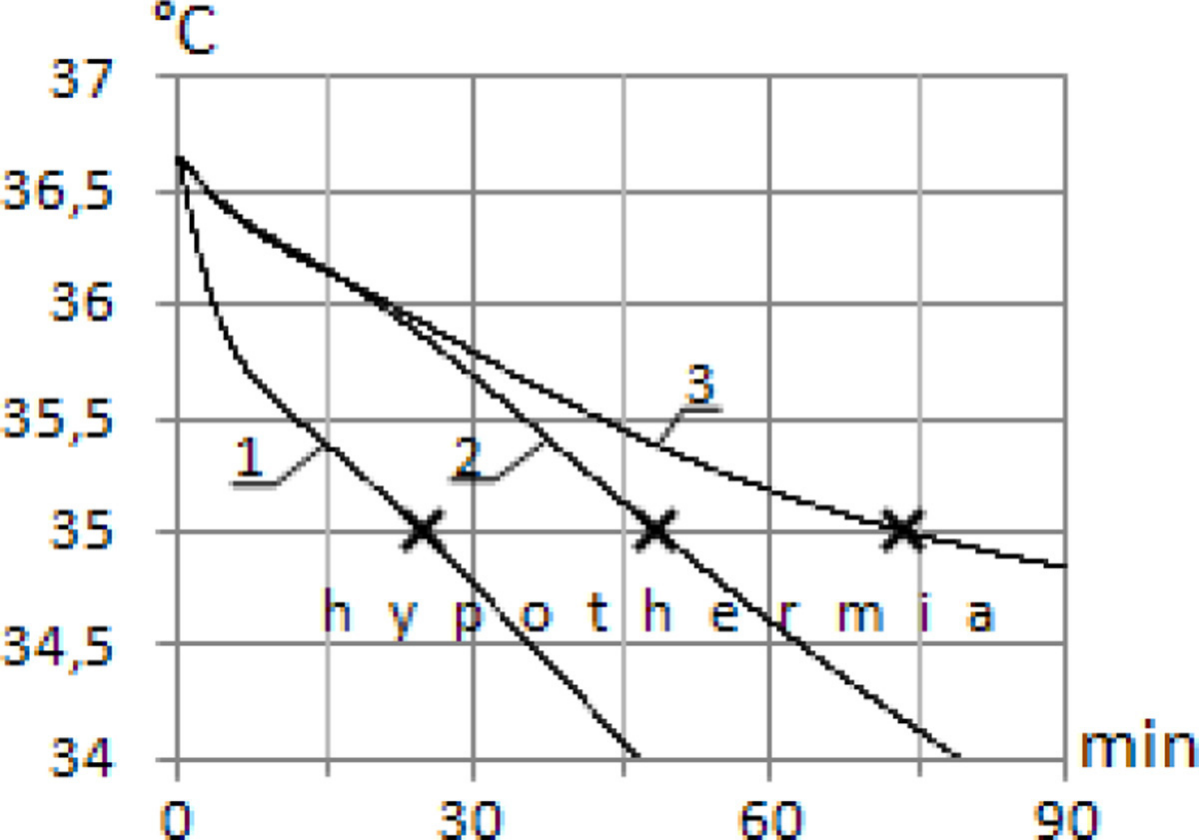
**Dynamics of brain temperature depending on immersion level: 1 - whole body immersion; 2 - head out of water; 3 - head, arms and hands out of water**. T_water _= 10°C, T_air _= 10°C.

Modelling (Figure 1) showed that brain temperature dropped to 35 °C during all cases of human immersion. But time characteristics were significantly different. During whole body immersion brain temperature decreased to 35 °C in 25 min, during head out immersion in 49 min, for head, arms and hands out of water in 74 min. Heat losses by water convection to this moment were 759 W (#1), 638 W (#2) and 465 W (#3). Modelling showed that internal organs temperature achieves critical value (35 °C) later. For full immersion it was 53 min, for head out of water 65 min and for head, arms and hands out 120 min. These data correlate with actual measurements of esophageal temperature in humans [3].

## Discussion

Modelling showed that evaluation of the safe time of human immersion in cold water depends on what part of body is immersed. Head out of water has principal effect on core cooling rate [4]. Modelling showed that the "safe time" increased two-fold during head-out immersion. Removing the arms and hands from the water decreased core cooling but not in proportion of their surface area; head surface is 8 % while arms and hands are 19 % of the body surface.

## Conclusion

Modelling prediction showed that thermoregulatory system is high sensitive to the immersion level of human in cold water. Surface out of water increases safe time of immersion. But head submersion has priority effect on core cooling. The results, when validated in all ranges, can be used for planning of rescue operations and development of protective clothing.

## References

[B1] Xu X Tikuisis P Thermoregulatory modeling for cold stress Comprehensive Physiology 2014 4 3 1057 1081 24944030 10.1002/cphy.c130047

[B2] Yermakova I Information platform for multicompartmental models of human temperature regulation Cybernetics and Computer Engineering 2013 174 81 91

[B3] Tikuisis P Giesbrecht GG Prediction of shivering heat production from core and mean skin temperatures European journal of applied physiology and occupational physiology 1999 79 3 221 229 10.1007/s004210050499 10048626

[B4] Pretorius T Lix L Giesbrecht GG Shivering heat production and body fat protect the core from cooling during body immersion, but not during head submersion: A structural equation model Computers in biology and medicine 2011 41 3 154 158 10.1016/j.compbiomed.2011.01.005 21295291

